# Comparison of PENTAX EB-1970UK and EB19-J10U ultrasound bronchoscopes for EBUS-TBNA in the diagnosis of mediastinal lymphadenopathy

**DOI:** 10.1186/s12890-025-04008-x

**Published:** 2025-11-19

**Authors:** Yingqi Zhang, Chuming Zhou, Cen Wu, Jian Wu, Peng Li

**Affiliations:** 1 Department of Pulmonary and Critical Care Medicine, Shengjing Hospital of China Medical University, No. 36, San Hao Street, Shenyang, 110004 Liaoning China; 2 Department of Anesthesiology, Shengjing Hospital of China Medical University, No. 36, San Hao Street, Shenyang, 110004 Liaoning China

**Keywords:** Endobronchial ultrasound, Transbronchial needle aspiration, Mediastinal lymphadenopathy, Elastography, Strain ratio

## Abstract

**Background and aim:**

Endobronchial ultrasound-guided transbronchial needle aspiration (EBUS-TBNA) is a widely used technique for evaluating mediastinal lymphadenopathy. However, limited data exist regarding the impact of different ultrasound bronchoscope models on procedural outcomes. This study assessed the technical performance, diagnostic efficacy, and safety of two PENTAX bronchoscopes—EB-1970UK and EB19-J10U—in EBUS-TBNA.

**Patients and methods:**

This retrospective study included patients with mediastinal lymphadenopathy who underwent EBUS-TBNA at Shengjing Hospital, China Medical University between January 2023 and March 2024. Patients were divided into two groups based on the bronchoscope used: EB-1970UK (n=73) and EB19-J10U (n=75). The groups were compared for specimen adequacy, complication rates, pathological positivity, diagnostic yield, and the predictive value of elastography in differentiating benign from malignant lymph nodes.

**Results:**

The specimen adequacy rate was significantly lower in the EB-1970UK group (89.77% vs. 97.03%, P < 0.05). The incidence of severe cough complications was higher in the EB-1970UK group (13.70% vs. 4.00%, P < 0.05). The pathological positivity rate (63.01% vs. 76.00%, P >0.05) and diagnostic yield (80.82% vs. 84.00%, P >0.05) were comparable. The strain ratio threshold for differentiating malignant from benign lymph nodes was 4.24 for EB19-J10Uand 2.115 for EB-1970UK, showing significant predictive value.

**Conclusion:**

Both bronchoscopes demonstrated high diagnostic accuracy and predictive value in elastography-assisted EBUS-TBNA for mediastinal lymphadenopathy. However, EB19-J10U provided better specimen adequacy and fewer severe cough complications, suggesting superior procedural efficiency and patient tolerance.

**Supplementary Information:**

The online version contains supplementary material available at 10.1186/s12890-025-04008-x.

## Introduction

Endobronchial ultrasound-guided transbronchial needle aspiration (EBUS-TBNA) is a minimally invasive and highly effective technique for evaluating mediastinal lymphadenopathy, particularly in the staging of lung cancer and differentiating between benign and malignant lymph nodes [1, 2]. Compared with traditional mediastinoscopy, EBUS-TBNA offers superior safety, lower procedural morbidity, and excellent diagnostic accuracy, making it a preferred first-line tool for mediastinal staging [1–3].

As EBUS-TBNA became more widely adopted, questions emerged about the optimal performance of the procedure and best conditions for a maximal diagnostic yield. Several factors influence the diagnostic yield of EBUS-TBNA, including needle size, the presence of rapid on-site cytologic evaluation (ROSE), and the approach used (transbronchial vs. transesophageal) [4–6]. Studies have shown that different needle gauges (19G, 21G, and 22G) offer similar diagnostic sensitivities, with the 19G needle providing better sample adequacy for molecular and immunohistochemical testing [7, 8]. The use of ROSE has been associated with fewer needle passes and improved sample adequacy. Additionally, the transesophageal approach using the EBUS bronchoscope (EUS-B-FNA) has been found to provide greater patient comfort with a similar diagnostic yield compared to the traditional transbronchial approach.

Despite these advancements, limited evidence exists on how different ultrasound bronchoscope models impact EBUS-TBNA outcomes. Variations in bronchoscope design, imaging resolution, and maneuverability may influence specimen adequacy, visualization quality, and complication rates. Notably, the PENTAX Medical Ultrasound Video Bronchoscope EB19-J10U is designed to improve diagnostic outcomes, featuring crystal clear ultrasound imaging to support reliable tissue acquisition [9, 10]. However, comparative studies evaluating different bronchoscope models remain scarce. Understanding these differences is crucial for optimizing EBUS-TBNA procedures and improving patient outcomes.​.

This study aims to compare the technical performance, diagnostic efficacy, and safety of two PENTAX ultrasound bronchoscope models—EB-1970UK and EB19-J10U—in patients undergoing EBUS-TBNA for mediastinal lymphadenopathy. By evaluating parameters such as specimen adequacy, complication rates, pathological positivity, diagnostic yield, and the predictive value of elastography in differentiating benign from malignant lymph nodes, this study seeks to provide evidence-based guidance on bronchoscope selection to enhance procedural efficiency and patient care.​.

## Patients and methods

### Study design and patients

This is a single-center retrospective study involving the collection and analysis of data from all patients who underwent EBUS-TBNA at Shengjing Hospital of China Medical University between January 2023 and March 2024 for mediastinal lymphadenopathy detected on computed tomography (CT). No formal sample size calculation was performed, as all eligible patients within the study period were included. Patients with incomplete clinical data, such as missing elastography images or pathological results, were excluded. Patients were divided into two groups according to the bronchoscope available at the time of the procedure: EB-1970UK (*n* = 73) and EB19-J10U (*n* = 75). The allocation was not randomized and depended on bronchoscope availability and operator scheduling, which may introduce selection bias. Follow-up clinical and imaging data were available for 122 patients (82.43%) at six months.

All patients fasted and refrained from water intake for 4–6 h prior to the procedure. The examination was conducted in a respiratory endoscopy room, with patients positioned in the supine position. Local airway anesthesia was administered using 2% lidocaine, and mediastinal lymph nodes were assessed using transnasal ultrasound bronchoscopy. Intravenous anesthesia was not used because, in our center, EBUS-TBNA is routinely performed under topical anesthesia only. This practice is consistent with local institutional protocols and allows for shorter recovery times, although it differs from BTS guidelines recommending intravenous sedation.

### EBUS-TBNA equipment

Two ultrasound bronchoscopy systems were used:

- EB- 1970UK group:
  - Ultrasound bronchoscope: EB-1970UK (PENTAX)
  - Endoscopic processor: EPK-i3000 (PENTAX)
  - Ultrasound scanner: Hi VISION Avius L (TOSHIBA, Japan)
- EB19- J10U group:
  - Ultrasound bronchoscope: EB19-J10U (PENTAX)
  - Endoscopic processor: EPK-i7010 (PENTAX)
  - Ultrasound scanner: ARIETTA 70 (TOSHIBA, Japan)

In both groups, transbronchial needle biopsies were performed using a dedicated 22-gauge needle (ECHO-HD-22-EBUS-P, COOK Medical).

### Acquisition and analysis of EBUS examination images

Before TBNA, the operator identified enlarged lymph nodes on preoperative CT scans, selected the appropriate lymph nodes for B-mode ultrasound imaging, and switched the imaging system to elastography mode. After obtaining stable elastic images, the operator used the equipment’s built-in functions to measure the strain rate (SR) of the target lymph nodes in real-time.

The specific process was as follows: 1) A region of interest (ROI) was drawn as large as possible within the target lymph node, and the device automatically calculated the SR of the tissue within this region, recorded as A.

2) A second ROI of similar size was drawn in adjacent normal tissue outside the target lymph node, ensuring that blood vessels and perivascular tissues were avoided in color Doppler mode. The SR of this normal tissue was recorded as B. 3) The SR ratio (B/A) was automatically calculated by the system.

### EBUS-TBNA procedure

Following B-ultrasound, Doppler blood flow imaging, and elastography, a bronchial ultrasound biopsy needle was inserted through the working channel of the bronchoscope, and puncture was performed under real-time ultrasound guidance. Each target lymph node was punctured 1–5 times, and the collected specimens were placed in formalin-containing specimen bottles for histopathological examination. While current ACCP guidelines recommend at least four punctures per station when malignancy or benign disease is suspected, in our practice the number of punctures was individualized based on lymph node size, patient tolerance, and operator judgment. In some patients, a lower number of punctures was sufficient to obtain adequate tissue while minimizing procedural discomfort.

### Evaluation of specimen adequacy

The adequacy of the obtained specimens was subjectively assessed by the operator based on visual inspection and documented as either satisfactory or insufficient. As this was a retrospective study, adequacy was not graded on a standardized visual analog scale but followed routine reporting practice in our center.

### Assessment of cough and biopsy site bleeding

The severity of cough and biopsy site bleeding was subjectively assessed by the operator throughout the procedure and documented in the final report as part of routine clinical data collection. Cough was classified into two levels (‘mild’ and ‘severe’). A formal visual analog scale was not applied, as the data were collected retrospectively.

### Criteria for pathological diagnosis

The cytological and histopathological results of EBUS-TBNA were classified into malignant, benign, and non-diagnostic categories. Malignant cases were defined by the presence of tumor cells or atypical cells indicative of malignancy, and EBUS-TBNA was considered to have a positive diagnostic value. Benign cases were characterized by the presence of granulomatous inflammation or other definitive benign lesions, confirming a benign diagnosis. Specimens containing coagulative necrosis, inflammatory cell infiltration, amorphous staining, cartilage fragments, fibrotic tissue, bronchial epithelial cells, or carbon deposits—without sufficient lymphoid tissue—were classified as non-diagnostic, and EBUS-TBNA was considered to have a negative diagnostic value.

Patients with benign or inconclusive EBUS-TBNA results underwent additional invasive procedures, including percutaneous lung biopsy, mediastinoscopy, or thoracoscopy, or received empirical treatment based on clinical diagnosis. Clinical and imaging follow-ups were conducted for at least six months to establish the final clinical diagnosis.

### Statistical analysis

All statistical analyses were performed using SPSS 22.0 (IBM Corp., Armonk, NY, USA). Continuous variables were expressed as mean ± standard deviation (SD). Categorical data were compared using the chi-square test, and if the assumptions for the chi-square test were not met, the continuity correction chi-square test was applied. Using pathological diagnosis as the gold standard, a receiver operating characteristic (ROC) curve was constructed to determine the area under the curve (AUC), sensitivity, specificity, positive predictive value (PPV), and negative predictive value (NPV) for different diagnostic indicators. A p-value of < 0.05 was considered statistically significant.

## Results

### Patient demographics

A total of 148 patients were evaluated (76 men and 72 women), aged 14 to 80 years. Among them, 73 patients were assigned to the EB-1970UK group and 75 to the EB19-J10U group. The demographic characteristics of the patients are summarized in Table 1.

**Table 1:** Demographics characteristics of all the studied patients

	Total	EB-1970UK n(%)	EB19-J10U n(%)	P-value
Age				0.189
< 60	73	40(54.79)	33(44.00)	
≥ 60	75	33(45.21)	42(56.00)	
Sex				0.408
Male	76	40(54.79)	36(48.00)	
Female	72	33(45.21)	39(52.00)	
Smoke				0.522
Yes	63	33(45.21)	30(40.00)	
No	85	40(54.79)	45(60.00)	
Total	148	73	75	

### Lymph node characteristics

A total of 189 lymph nodes were sampled in 148 patients, with an average of 1.27 lymph nodes per patient. Pathological specimens were successfully obtained in all cases, yielding a success rate of 100%. Lymph nodes were classified according to the latest International Staging System for malignant lymph nodes. This classification was applied for anatomical mapping purposes, regardless of whether the final diagnosis was malignant or benign. The proportion of specimens reaching diagnostic adequacy was higher in the EB19-J10U group compared to the EB-1970UK group. The lymph node characteristics of the patients are summarized in Table 2 and in Supplementary Table 1.

**Table 2:** Characteristics of the lymph nodes evaluated (summary results; full details provided in supplementary table S1)

Measure	Total *n*(%)	EB-1970UK *n*(%)	EB19-J10U *n*(%)	*P*-value
Number of LNs	189	88	101	—
Specimen adequacy	177 (93.65%)	79 (89.77%)	98 (97.03%)	0.041
Pathological positive LNs	142 (75.13%)	65 (73.86%)	77 (76.24%)	0.706

### Pathological and clinical diagnosis

A total of 103 cases of pathological positive diagnoses were obtained through EBUS-TBNA, resulting in diagnostic accuracy of 69.59% (103/148) for benign and malignant diseases. The sensitivity of EBUS-TBNA for diagnosing malignant diseases was 98.41% (62/63), while the sensitivity for non-malignant diseases was 69.49% (41/59).

Among non-malignant diseases, EBUS-TBNA successfully diagnosed 24 out of 40 cases of sarcoidosis (60%) and 3 out of 4 cases of tuberculosis (75%). Of the sarcoidosis cases not definitively diagnosed by EBUS-TBNA, four patients were diagnosed with non-caseating granulomas through bronchial mucosal biopsy and confirmed after effective glucocorticoid treatment. The remaining 12 patients were followed long-term, and sarcoidosis was considered the most likely clinical diagnosis. For tuberculosis, EBUS-TBNA diagnosed 3 out of 4 cases using a combination of histology, TB-PCR, and other diagnostic methods. The remaining case had positive tuberculosis-related laboratory tests, and clinical follow-up strongly suggested tuberculosis, which was ultimately confirmed following effective diagnostic anti-tuberculosis treatment.

**Fig. 1:**
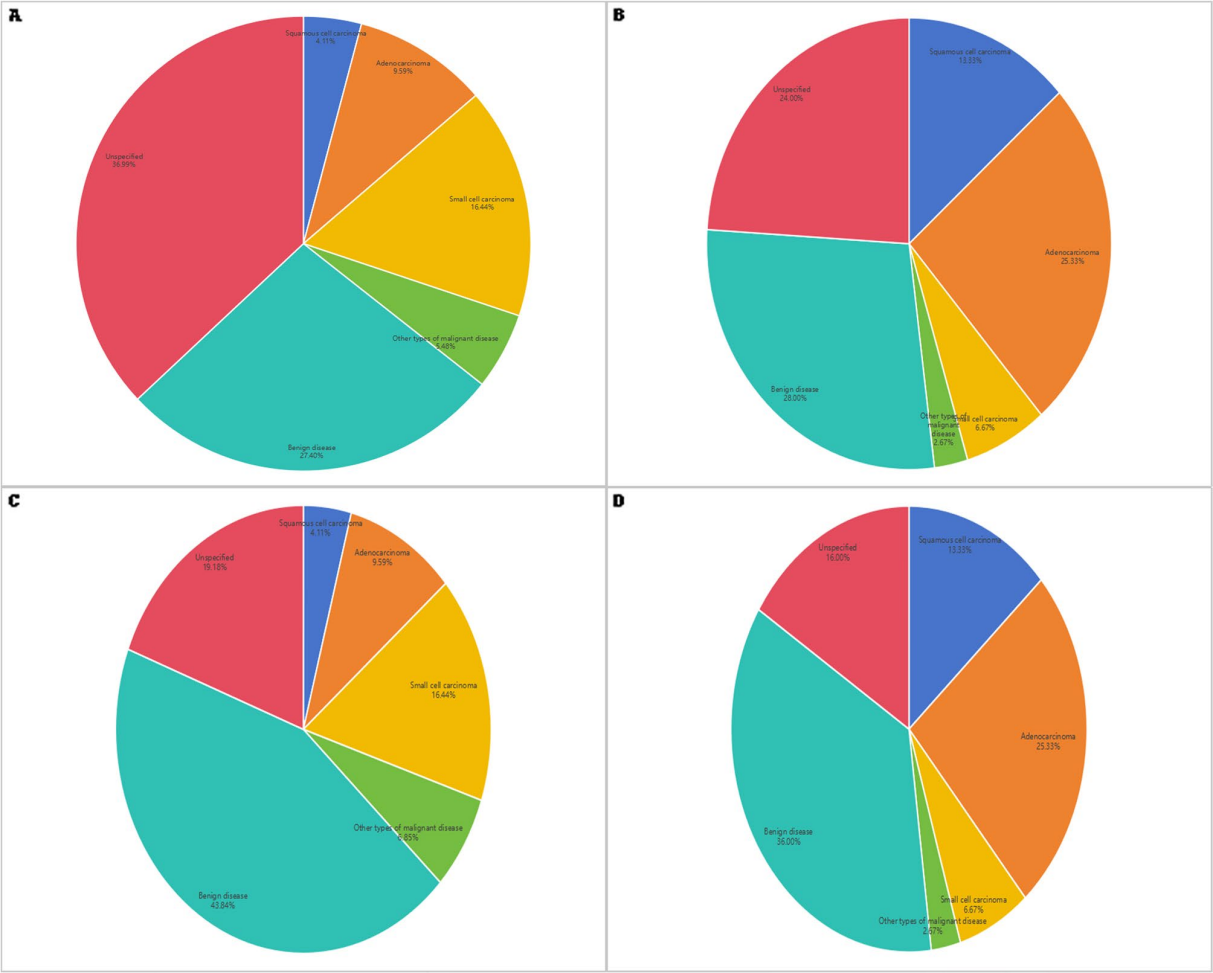
Pathological and clinical diagnoses in patients undergoing EBUS-TBNA with EB-1970UK and EB19-J10U bronchoscopes. **A** Distribution of pathological diagnoses in the EB-1970UK group (*n* = 73) (**B**) Distribution of pathological diagnoses in the EB19-J10U group (*n* = 75) (**C**) Final clinical diagnoses in the EB-1970UK group after integration of pathological results and clinical follow-up (**D**) Final clinical diagnoses in the EB19-J10U group after integration of pathological results and clinical follow-up The figure illustrates the comparative distribution of malignant and benign conditions (including sarcoidosis, tuberculosis, non-specific inflammation, and carcinoma subtypes) between the two groups

Among the 148 patients, 122 received a clinical diagnosis, including 26 cases of adenocarcinoma, 13 cases of squamous cell carcinoma, and 17 cases of small cell carcinoma. One patient had lymph node pathology initially showing lymphocytes, but was later diagnosed with lymphoma after a puncture biopsy of enlarged lymph nodes one year later. Benign conditions included 40 cases of sarcoidosis, 4 cases of tuberculosis, 21 cases of non-specific inflammation, and 1 case of mediastinal abscess. A total of 26 patients did not receive a definitive diagnosis due to the absence of further examinations or ongoing follow-up. Detailed pathological and clinical diagnosis results are presented in Table 3, supplementary Tables 2 and Figs. 1.

**Table 3:** Comparison of pathological and clinical diagnoses between groups (summary results; detailed subtype breakdowns provided in supplementary table S2)

Outcome	EB-1970UK	EB19-J10U	*P*-value
Pathological diagnostic rate	46 (63.01%)	57 (76.00%)	0.086
Clinical diagnostic rate	59 (80.82%)	63 (84.00%)	0.611

### Safety analysis of EBUS-TBNA

The most common procedural complications associated with EBUS-TBNA were cough and minor bleeding at the puncture site. One patient in the 1970UK group experienced active bleeding at the puncture site during the procedure, classified as moderate in severity. The bleeding was successfully controlled with local endoscopic epinephrine spraying and intravenous infusion of pituitrin. Some patients experienced transient oxygen desaturation due to severe coughing; however, symptoms resolved rapidly after pausing the procedure and administering lidocaine for airway surface anesthesia, leading to immediate cough relief and restoration of oxygen levels. The incidence of severe cough-related complications was higher in the EB-1970UK group compared to the EB19-J10U group. Severe cough-related complications were defined as episodes of persistent coughing during the procedure that caused transient oxygen desaturation or required interruption of the procedure for airway management. No serious complications, such as pneumothorax, hemopneumothorax, infection, or laryngeal spasm, were observed. The distribution of puncture-related complications between the RB-1970UK and EB19-J10U groups is presented in Table 4.

**Table 4:** Puncture complications in EB-1970 UK group and EB19-J10U group

	Total	EB-1970UK *n*(%)	EB19-J10U *n*(%)	*P*-value
Cough				0.037
Mild	135	63(86.30)	72(96.00)	
Severe	13	10(13.70)	3(4.00)	
Biopsy site bleeding				---
Less	147	72(98.63)	75(100.00)	
Moderate	1	1(1.37)	0	

### Diagnostic value of quantitative elastography in differentiating benign and malignant mediastinal and hilar lymph nodes

The diagnostic performance of ultrasound elastography in differentiating benign and malignant mediastinal and hilar lymph nodes was assessed using the strain ratio (SR) index. Table 5 presents the cut-off values and diagnostic accuracy of elastography indices for the EB-1970UK and EB19-J10U groups. The cut-off value for the EB19-J10U group was 4.24, while the cut-off value for the EB-1970UK group was 2.115. Both cut-off values were statistically significant (*p* < 0.05), indicating that elastography provides valuable diagnostic differentiation between benign and malignant lymph nodes.

**Table 5:** Diagnostic value of elastography indexes for benign and malignant lymph nodes in EB- 1970 UK group and EB19-J10U group

B/A	Cut-off value	Accuracy (%)	Sensitivity (%)	Specificity (%)	PPV (%)	NPV (%)	AUC	*p*-value malignant vs. benign
EB-1970UK	2.115	67.69	0.586	0.75	65.38%	69.23%	0.675	0.016
EB19- J10U	4.24	67.53	0.537	0.833	78.57	61.22	0.702	0.002

## Discussion

Endobronchial ultrasound-guided transbronchial needle aspiration (EBUS-TBNA) is a well-established, minimally invasive procedure for evaluating mediastinal lymphadenopathy, particularly in lung cancer staging and diagnosing benign and malignant lymph node diseases [11, 12]. Despite its widespread use, variations in bronchoscope design may influence procedural outcomes, including specimen adequacy, diagnostic yield, and complication rates. This study compared two PENTAX ultrasound bronchoscope models, EB-1970UK and EB19-J10U, assessing their performance in terms of tissue acquisition, safety profile, and diagnostic value of elastography in distinguishing benign from malignant lymph nodes.

Our findings indicate that both bronchoscope models demonstrated high diagnostic accuracy, with comparable pathological positivity rates (63.01% vs. 76.00%, *p* > 0.05) and diagnostic yields (80.82% vs. 84.00%, *p* > 0.05). However, significant differences were observed in specimen adequacy and complication rates. The EB19-J10U model achieved a significantly higher satisfactory specimen acquisition rate (97.03% vs. 89.77%, *p* < 0.05), suggesting that its enhanced design features contribute to improved sample retrieval. Similar results have been reported in studies evaluating the impact of bronchoscope modifications on procedural success [13, 14]. The improved performance of the EB19-J10U may be attributed to its increased curvature radius and optimized needle positioning, which reduce deformation during puncture and enhance tissue collection efficiency.

### Safety profile and complications

Procedural safety is a major concern in EBUS-TBNA. The most common adverse events in our study were cough and minor biopsy site bleeding, consistent with previous reports [15, 16]. The incidence of severe cough was significantly lower in the EB19-J10U group (4.00% vs. 13.70%, *p* < 0.05). While this difference may in part be attributed to the larger working channel diameter of the EB19-J10U (2.2 mm), which allows more efficient suctioning of secretions and reduces airway irritation, it should also be noted that cough severity is strongly influenced by the type of anesthesia used. In our study, all procedures were performed under topical lidocaine anesthesia without intravenous sedation, which differs from BTS recommendations. The absence of IV sedation may have contributed to the higher cough rates overall and should be considered when interpreting these findings. Additionally, the improved ergonomics of the EB19-J10U model facilitate smoother needle insertion, minimizing procedural distress for both the operator and the patient. From the operator’s perspective, enhanced maneuverability reduces technical difficulty, while from the patient’s perspective, smoother insertion may reduce procedure-related discomfort. Prior research has highlighted bronchoscope maneuverability and suction capacity as key factors influencing patient tolerance during EBUS-TBNA [17].

Notably, no major complications such as pneumothorax, hemopneumothorax, infection, or laryngeal spasm occurred in either group, aligning with published safety profiles for EBUS-TBNA [18, 19]. While severe bleeding was rare, one case in the EB-1970UK group required local hemostatic intervention with epinephrine and intravenous pituitrin, reflecting the importance of real-time bleeding control strategies in EBUS-TBNA [20].

### Diagnostic value of elastography in lymph node evaluation

Ultrasound elastography, a non-invasive imaging technique, assesses tissue stiffness to differentiate between benign and malignant lymph nodes. In this study, the strain ratio (SR) cut-off value was significantly higher in the EB19-J10U group (4.24 vs. 2.115, *p* < 0.05). While these findings suggest potential value in differentiating malignant from benign lymph nodes, the sensitivity and specificity were modest and should be interpreted with caution. Our results are broadly consistent with previous elastography studies, which have reported SR cut-offs ranging from 2.5 to 6.5 [21, 22], but further validation in larger cohorts is required before firm conclusions can be drawn.

The higher AUC value (0.784) in the EB19-J10U group, compared to the EB-1970UK group (0.684), suggests improved diagnostic reliability. A recent meta-analysis concluded that SR values above 3.0 yield high sensitivity and specificity in predicting malignancy, supporting our results [23]. Additionally, the EB19-J10U model enables direct SR calculations from B-mode images, reducing potential bias from overlaying elastographic data and improving diagnostic confidence [24].

### Comparison with existing literature and clinical implications

Several studies have emphasized the importance of bronchoscope design in optimizing procedural efficiency and diagnostic outcomes. For instance, a randomized trial comparing Olympus and Fujifilm EBUS bronchoscopesdemonstrated that enhanced imaging resolution and improved needle angulation positively influence sample adequacy and diagnostic yield [25, 26]. Our findings extend this understanding to PENTAX bronchoscopes, highlighting that the EB19-J10U offers a more favorable procedural profile than the EB-1970UK in terms of specimen adequacy, elastography performance, and patient tolerance.

From a clinical perspective, these results suggest that choosing an optimized bronchoscope model can enhance procedural efficiency, reduce patient discomfort, and improve the overall diagnostic workflow in mediastinal lymphadenopathy evaluation. Future studies should explore real-time elastography-guided biopsy strategies and investigate the role of advanced ultrasound technologies in refining EBUS-TBNA techniques.

### Limitations

This study has several limitations. First, it was a retrospective, single-center study, which may limit the generalizability of the findings. Second, patient allocation to bronchoscope groups was not randomized, and thus selection bias cannot be excluded. Third, sample adequacy, cough severity, and bleeding severity were subjectively assessed by the operator, which may introduce reporting bias. Finally, the modest sample size and limited follow-up duration may affect the strength of the conclusions. Future multicenter prospective studies with standardized assessment tools are warranted to confirm these results.

## Conclusion

In conclusion, both PENTAX EB-1970UK and EB19-J10U bronchoscopes demonstrated high diagnostic accuracy for mediastinal lymphadenopathy. The EB19-J10U showed somewhat higher specimen adequacy and fewer severe cough events, suggesting a more favorable procedural profile. Elastography-assisted SR measurements with both bronchoscopes provided useful, though modest, diagnostic value. These results should be interpreted with caution and warrant confirmation in larger prospective studies.

## Supplementary Information

Supplementary Material 1.

## Data Availability

The datasets generated and/or analyzed during the current study are available from the corresponding author upon reasonable request.

## Abbreviations

EBUS-TBNA: Endobronchial ultrasound–guided transbronchial needle aspiration
ROC: Receiver operating characteristic
AUC: Area under the curve
SR: Strain ratio
PPV: Positive predictive value
NPV: Negative predictive value
CT: Computed tomography
SD: Standard deviation
