# Supplementary material for: Comparison of PENTAX EB-1970UK and EB19-J10U ultrasound bronchoscopes for EBUS-TBNA in the diagnosis of mediastinal lymphadenopathy
Source: BMC Pulm Med. 2025 Nov 19;25:538. doi: 10.1186/s12890-025-04008-x (PMC12628913; doi:10.1186/s12890-025-04008-x)
Supplement: Supplementary file 1 — Supplementary Material 1. [file 12890_2025_4008_MOESM1_ESM.docx]

## Supplementary Table S1. Detailed characteristics of lymph nodes evaluated in the study.

| LN station | Total n(%) | EB-1970UK n(%) | EB19-J10U n(%) | P-value |
| --- | --- | --- | --- | --- |
| 2R | 3 (1.59) | 2 (2.27) | 1 (0.99) | 0.904 |
| 3P | 2 (1.06) | 0 | 2 (1.98) | - |
| 4R | 43 (22.75) | 18 (20.45) | 25 (24.75) | 0.482 |
| 4L | 4 (2.12) | 3 (3.41) | 1 (0.99) | - |
| 7 | 82 (43.39) | 33 (37.5) | 49 (48.51) | 0.127 |
| 10R | 4 (2.12) | 3 (3.41) | 1 (0.99) | - |
| 10L | 2 (1.06) | 1 (1.14) | 1 (0.99) | - |
| 11R | 29 (15.34) | 19 (21.59) | 10 (9.9) | 0.026 |
| 11L | 15 (7.94) | 8 (9.09) | 7 (6.93) | 0.584 |
| 12R | 2 (1.06) | 1 (1.14) | 1 (0.99) | - |
| 12L | 2 (1.06) | 0 | 2 (1.98) | - |
| 13L | 1 (0.53) | 0 | 1 (0.99) | - |

LN=Lymph nodes

## Supplementary Table S2. Detailed pathological and clinical diagnoses of patients.

| Diagnosis | EB-1970UK | EB19-J10U | P-value |
| --- | --- | --- | --- |
| Malignant disease | 26 (35.62%) | 36 (48.00%) | 0.127 |
| Squamous cell carcinoma | 3 | 10 | — |
| Adenocarcinoma | 7 | 19 | — |
| Small cell carcinoma | 12 | 5 | — |
| Neuroendocrine carcinoma | 1 | 0 | — |
| Lymphoma | 0 | 0 | — |
| Mesenchymal tumors | 2 | 0 | — |
| Serous carcinoma | 1 | 0 | — |
| Malignancy of unknown type | 0 | 2 | — |
| Benign disease | 20 (27.40%) | 21 (28.00%) | 0.935 |
| Sarcoidosis | 11 | 13 | — |
| Tuberculosis | 2 | 1 | — |
| Non-specific inflammation | 7 | 7 | — |
| Mediastinal abscess | 0 | 0 | — |
| Unspecified | 27 (36.99%) | 18 (24.00%) | — |
